# Supplementary material for: The nucleus accumbens shell regulates hedonic feeding via a rostral hotspot
Source: eLife. 2026 May 21;14:RP108639. doi: 10.7554/eLife.108639 (PMC13193714; doi:10.7554/eLife.108639)
Supplement: Supplementary file 1. [file elife-108639-supp1.docx]

**Stard5-2A-Flp0 KI template sequence**

Homology arm

RAKR

T2A

FlpO

TATCATCCACAACAAGGAGGCTGAGAGGAGTCCTGGGCACTGAATCTAGAGCAGGAGTAGAAGTGACCACCATGTCTGGACCACTTCTGCTGGTCATGGCTCTGAGCTAGATGCTTCCATGGTACATATCTAACCCGCATCTGCTATTTCTTCAGGGATCCCAACAAAACCAACCTGGTCACATTCTTTCAGACAGACCTGAGTGGCTACCTCCCTCAGAGTGTGGTGGACTCCTTCTTCCCTCGAAGCATGGCTGAGTTCTACCCCAACCTTCAGAAGGCAGTGAGGAAGTTCCATCACAGGGCAAAGAGGgagggcagaggaagtcttctaacatgcggtgacgtggaggagaatcccggccctatggctcctaagaagaagaggaaggtgatgagccagttcgacatcctgtgcaagaccccccccaaggtgctggtgcggcagttcgtggagagattcgagaggcccagcggcgagaagatcgccagctgtgccgccgagctgacctacctgtgctggatgatcacccacaacggcaccgccatcaagagggccaccttcatgagctacaacaccatcatcagcaacagcctgagcttcgacatcgtgaacaagagcctgcagttcaagtacaagacccagaaggccaccatcctggaggccagcctgaagaagctgatccccgcctgggagttcaccatcatcccttacaacggccagaagcaccagagcgacatcaccgacatcgtgtccagcctgcagctgcagttcgagagcagcgaggaggccgacaagggcaacagccacagcaagaagatgctgaaggccctgctgtccgagggcgagagcatctgggagatcaccgagaagatcctgaacagcttcgagtacaccagcaggttcaccaagaccaagaccctgtaccagttcctgttcctggccacattcatcaactgcggcaggttcagcgacatcaagaacgtggaccccaagagcttcaagctggtgcagaacaagtacctgggcgtgatcattcagtgcctggtgaccgagaccaagacaagcgtgtccaggcacatctactttttcagcgccagaggcaggatcgaccccctggtgtacctggacgagttcctgaggaacagcgagcccgtgctgaagagagtgaacaggaccggcaacagcagcagcaacaagcaggagtaccagctgctgaaggacaacctggtgcgcagctacaacaaggccctgaagaagaacgccccctaccccatcttcgctatcaagaacggccctaagagccacatcggcaggcacctgatgaccagctttctgagcatgaagggcctgaccgagctgacaaacgtggtgggcaactggagcgacaagagggcctccgccgtggccaggaccacctacacccaccagatcaccgccatccccgaccactacttcgccctggtgtccaggtactacgcctacgaccccatcagcaaggagatgatcgccctgaaggacgagaccaaccccatcgaggagtggcagcacatcgagcagctgaagggcagcgccgagggcagcatcagataccccgcctggaacggcatcatcagccaggaggtgctggactacctgagcagctacatcaacaggcggatcTGACAGTCGCCTATTGGCAGGGATTGCCTATGGTCCTTCAGGAACTCCAGCTGTTGAGCCTCAAAGGACACGGAAGAGCTGCTGAGGCCTTTTGAGATGCCATAGTTAACAGCAAATCTCAGGGTTCCATTCCAGAGGAGCAGCCCATCCCCCCACACCACCCTCCGTCCTCTTGGGCTTGGTGTGGAAGGAGCTAACCACTAGCTTGCCGTGGTGCACACATAGGCCTGGCAGGCCAGGGCAAGTCCCTCTATGGCCACCTGAGAGATGGCTCACGTACTCTTTGATTCCTTGTGGTCC
