# Supplementary material for: The nucleus accumbens shell regulates hedonic feeding via a rostral hotspot
Source: eLife. 2026 May 21;14:RP108639. doi: 10.7554/eLife.108639 (PMC13193714; doi:10.7554/eLife.108639)
Supplement: Supplementary file 2. [file elife-108639-supp2.docx]

**Python code in Pynapse for unpredicted reward and unpredicted shock tasks and Optogenetic stimulation during feeding task**

**Unpredicted Reward Experiment**

The experiment is coded in Pynapse (Python-based code in the Synapse software from Tucker Davis Technologies), driving iCON behavioral control units.

Experiment: The session lasts about 30 min. Time-out period of 3 min (180s) at start. Trial duration: 10 s (i.e. lickometer made available). Trial number: 30 times. Inter-trial interval: random intervals, 30 to 50 s. A succesful trial is defined as a trial for which the animal licked the lickometer at least once. For succesful trials, the number of licks is quantified. A lick is defined (within Synapse) as at least 3ms interacting with the lickometer.

Pynapse Code:

## Pynapse Source #

import random

import tdt

import numpy

class Always: #StateID = 0

def s_Mode_recprev():

p_Rig.HouseFan.turnOn()

p_Rig.HouseLight.turnOn()

p_State.switch(PreTrial)

class PreTrial: # StateID = 1010

def s_State_enter():

p_State.setTimeout(180, Trial1)

class Trial1: # StateID = 1020

def s_State_enter():

p_Rig.RoundLED.turnOff()

p_Metric.Count.inc()

p_Metric.FirstLick.write(0)

random.seed(56)

p_Metric.ITI_List = []

for n in range(0,30): #edit to change number of trials or inter-trial intervals (ITI)

p_Metric.ITI = random.randrange(30,50,1) #edit to change range of ITI

p_Metric.ITI_List.append(p_Metric.ITI)

#print(p_Metric.ITI_List)

print("Succesful trials = ", p_Metric.SuccessfulTrials.read())

p_State.cancelTimeout()

if p_Metric.Count.read() <= len(p_Metric.ITI_List) and p_Metric.Trials.read()<=30: #here, 30=total number of trials (=total number of ITI)

print("Seconds before next trial = ",p_Metric.ITI_List[p_Metric.Count.read()-1])

p_State.setTimeout(p_Metric.ITI_List[p_Metric.Count.read()-1],Trial2)

else:

p_State.switch(PostTrial)

'''#TIMEOUT ONLY FOR TRAINING! REMOVE FOR ACTUAL EXPERIMENT!!

def s_Lick_rise():

p_Metric.ExtraLicks.inc()

if p_Metric.ExtraLicks.read()==5: #if more than 5 licks outside the reward window, timeout initiated

p_Metric.Timeouts.inc()

print("lick detected outside reward window; timeout initiated")

p_State.setTimeout(p_Metric.ITI_List[p_Metric.Count.read()-1]+60, Trial2) #timeout=60s+whatever the current ITI is

'''

class Trial2: # StateID = 1030

def s_State_enter():

p_Rig.Lickometer.fire()

p_Metric.Trials.inc()

print("reward available")

print("trial number = ", p_Metric.Trials.read())

p_Metric.ExtraLicks.write(0)

p_State.setTimeout(10, Trial1)

def s_Lick_active():

print("lick")

p_Metric.Licks.inc() #at end of session, Licks=total number of valid and invalid licks from all reward windows

p_Metric.FirstLick.inc()

if p_Metric.FirstLick.read()==1:

p_Rig.RoundLED.turnOn()

p_Metric.SuccessfulTrials.inc()

print("first lick")

def s_Lick_fall():

print("no lick")

def s_Lick_fail():

print("invalid lick")

p_Metric.InvalidLicks.inc() #at end of sesson, InvalidLicks=total number of invalid licks from aa reward windows

class PostTrial: # StateID = 1040

def s_State_enter():

p_State.setTimeout(180, EndRecording)

class EndRecording: # StateID = 1050

def s_State_enter():

p_Rig.HouseFan.turnOff()

p_Rig.HouseLight.turnOff()

syn.setModeStr('Idle')

**Unpredicted Shock Experiment**

The experiment is coded in Pynapse (Python-based code in the Synapse software from Tucker Davis Technologies), driving iCON behavioral control units.

Experiment: Time-out period of 3 min (180s) at start, followed by a 1 s shock delivered 10 times at random intervals between 40 and 80 seconds

Pynapse Code:

# Pynapse Source #

import random

class Always: #StateID = 0

def s_Mode_recprev():

p_Rig.HouseFan.turnOn()

p_Rig.HouseLight.turnOn()

p_State.switch(PreTrial)

class PreTrial: # StateID = 1010

def s_State_enter():

p_State.setTimeout(180, InterTrialInterval)

class InterTrialInterval: # StateID = 1020

def s_State_enter():

p_Metric.Count.inc()

random.seed(98)

iti_list = []

for n in range(0,10):

iti=random.randrange(40,80, 1)

iti_list.append(iti)

#print(iti_list)

p_State.cancelTimeout()

if p_Metric.Count.read() <= len(iti_list) and p_Metric.Shocks.read()<=10:

print("seconds before next shock = ", iti_list[p_Metric.Count.read()-1])

p_State.setTimeout(iti_list[p_Metric.Count.read()-1],Shock)

else:

p_State.switch(PostTrial)

class Shock: # StateID = 1030

def s_State_enter():

p_Rig.GridShock.fire()

print("shock given; trial number = ",p_Metric.Count.read())

p_Metric.Shocks.inc()

p_State.switch(InterTrialInterval)

class PostTrial: # StateID = 1040

def s_State_enter():

p_State.setTimeout(180, EndRecording)

class EndRecording: # StateID = 1050

def s_State_enter():

p_Rig.HouseFan.turnOff()

p_Rig.HouseLight.turnOff()

syn.setModeStr('Idle')

**Optogenetic stimulation during reward task**

Reward is available at all times. Optogenetic stimulation, triggered by TDT (5 minutes, 20 Hz)

## Pynapse Source #

#this code has 5 blocks: trial 1 opto STIM, trial 2 NO stim, trial 1 opto STIM, trial 2 NO stim, trial 5 OPTO STIM

#each block is 5 min long

import random

import tdt

import numpy

class Always: #StateID = 0

def s_Mode_recprev():

p_Rig.HouseFan.turnOn()

p_Rig.HouseLight.turnOn()

p_Timer.MyTimer.turnOn()

p_State.switch(PreTrial)

class PreTrial: # StateID = 1010

def s_State_enter():

print("Baseline 2 s")

p_Metric.TrialCounter.write(0) # Initialize a counter to track the number of trials

p_State.setTimeout(2, Trial1)

class Trial1: # StateID = 1030

def s_State_enter():

p_Output.StartLaser.fire() # Turn on the laser 20 Hz stim continous

p_Metric.Trials.inc()

print("Trial 1: OPTO stimulation, trial Nr.", p_Metric.Trials.read())

p_State.setTimeout(300, SwitchTrial) # After 5 minutes (300 seconds), switch to next trial

def s_Lick_active():

print("lick")

p_Metric.Licks.inc()

class Trial2: # StateID = 1031

def s_State_enter():

p_Metric.Trials.inc()

print("Trial 2: NO opto stim 5 min, trial Nr. ", p_Metric.Trials.read())

p_State.setTimeout(300, SwitchTrial) # After 5 minutes (300 seconds), switch to next trial

def s_Lick_active():

print("lick")

p_Metric.Licks.inc()

class SwitchTrial: # StateID = 1040

def s_State_enter():

current_trial = p_Metric.TrialCounter.read()

if current_trial < 4: # Check if there are more trials to run

if current_trial % 2 == 0: # If the trial count is even, switch to Trial 2

p_State.switch(Trial2)

else: # If the trial count is odd, switch to Trial 1 (opto stim)

p_State.switch(Trial1)

p_Metric.TrialCounter.inc()

else:

p_State.switch(PostTrial) # After 5 trials (Trial 1 Trial 2 Trial 1 Trial 2 Trial 1), end the session

class PostTrial: # StateID = 1050

def s_State_enter():

#p_Rig.Speaker2.fire()

print("POST TRIAL: 5 seconds left!!!!")

p_State.setTimeout(5, EndRecording)

class EndRecording: # StateID = 1060

def s_State_enter():

p_Rig.HouseFan.turnOff()

p_Rig.HouseLight.turnOff()

syn.setModeStr('Idle')
